# Supplementary material for: Associations of Occupational Heat Stress and Noise Exposure with Carotid Atherosclerosis among Chinese Steelworkers: A Cross-Sectional Survey
Source: Int J Environ Res Public Health. 2021 Dec 21;19(1):24. doi: 10.3390/ijerph19010024 (PMC8751136; doi:10.3390/ijerph19010024)
Supplement: Supplementary file 1 [file ijerph-19-00024-s001.zip › ijerph-1469403-supplementary.pdf]

Supplementary file

Associations of occupational heat stress and noise exposure with carotid atherosclerosis among Chinese steelworkers: a cross-sectional survey

Lihua Wang, Miao Yu, Shengkui Zhang, Xiaoming Li, Juxiang Yuan

Department of Epidemiology and Health Statistics, School of Public Health, North China University of Science and Technology, Tangshan, Hebei Province, China

Correspondence to
Professor Juxiang Yuan;
yuanjx@ncst.edu.cn

Lihua Wang and Miao Yu contributed equally to this paper

Assessment of covariates

The level of education was divided into three categories: “primary or middle”, “high school or college”, and “university and above”.

Smoking status was evaluated from self-reported information, mainly including the age at starting smoking and the number of cigarettes consumed per day, and was divided into never smokers (who had never smoked in their lifetime); ever smokers (who had quit cigarettes earlier than 12 months before) and current smokers (who had regularly consumed ≥1 cigarette/day for at least the past 12 months) [1]. Current smokers were estimated in pack-years, and were further grouped into three categories: < 11 pack-years, 11-20 pack-years and > 20 pack-years [2].

Drinking status was evaluated from self-reported information, mainly including the amount and frequency of alcohol consumed per week and was divided into never drinkers (never or almost never drank alcohol in the past 12 months and had not drunk in most weeks in any past year); ever drinkers (did not drink alcohol in most weeks in the past 12 months but did so in some past year(s)) and current drinkers (drank alcohol usually at least once a week over the past 12 months). For current drinkers, the frequency of drinking status (days/week), usually the average amount of alcohol consumed (g), and types of beverages were recorded. Alcohol content across different types of beverages in China was assessed as follows: strong spirits 53%, weak spirits 38%, rice wine 15%, grape wine 12%, and beer 4%. Based on the beverage type, amount consumed, and frequency, we were able to derive the amount of pure alcohol (g/week) consumed per week. Current drinkers were further grouped into those drinking <140 g/week and ≥140 g/week [3].

Dietary patterns were assessed based on the DASH diet score [4]. Dietary patterns were assessed based on the DASH diet score, which was based on eight foods and nutrients that were either emphasized or deemphasized in the DASH-style diet [5]. Each component was scored from 1 to 5 points according to fifths of intake, with 5 being the best score for higher intake of vegetables, fruits, nuts and legumes, whole grains, and low fat dairy products and for lower intake of sugar sweetened drinks, red and processed meats, and sodium.

The calculation of metabolic equivalents was based on the International Physical Activity Questionnaire (IPAQ) [6]. The workers with metabolic equivalent task (MET) [min/week] values < 600, 600-3000 and > 3000 were classified as having a low, moderate, and high level of physical activity respectively.

The duration of sleep was the weighted averages of sleep on working days and rest days.

The insomnia assessment was estimated using the 8-item Athens Insomnia Scale (AIS) [7]. The first 5 items evaluate difficulty with sleep induction, awakening during the night, early-morning awakening, total sleep time, and overall quality of sleep; the last 3 items focus on sense of well-being, overall functioning, and sleepiness during daytime. Each item was scored on a 4-point Likert scale from 0 (no problem at all) to 3 (a very serious problem), with the total score ranging from 0 to 24. A score of ≥ 6, which being the widely accepted cut-off value for insomnia.

Standard study protocols were used to train qualified physicians and nurses prior to this survey. Height and weight were measured three times each. The participants stood upright and barefoot in light clothes. The height and weight data that were ultimately used for analysis were accurate to 0.1 cm and 0.1 kg. Body mass index (BMI) was defined as body weight (kg) divided by the square of the body height (m^2).

Blood pressure measurements were performed three times five-minute intervals using an electronic sphygmomanometer (OMRON, HBP-1100, China), and the participants were required to rest for more than ten minutes. Finally, the mean was obtained for analysis. Hypertension was defined as current systolic blood pressure ≥140 mmHg, or diastolic blood pressure ≥90 mmHg, or if the patient was receiving antihypertensive therapy.

Participants were required to fast overnight before the health examination and blood collection. Participants' anterior elbow vein blood was collected and centrifuged at room temperature (3000 r/min, 15 minutes) immediately. All blood samples were tested in the central laboratory of Tangshan Hongci Hospital Laboratory using automatic biochemical analysers (mindrary, BS-800, China) within four hours.

Diabetes was defined as fasting blood glucose ≥7.0 mmol/L or if the patient was receiving hypoglycemic therapy.

Total cholesterol (TC) ≥6.22 mmol/L or low-density lipoprotein (LDL-C) ≥4.11 mmol/L or high-density lipoprotein (HDL-C) ≤1.04 mmol/L or triglycerides (TG) ≥2.32 mmol/L, or patients undergoing lipid-lowering therapy were considered to demonstrate dyslipidemia.

We also recorded whether the workers had taken antihypertensive, antidiabetic, or lipid-lowering drugs in the last two weeks.

Table of contents

Table S1 Basic characteristics of participants according to sex

Variables	Total	Male	Female	*p*-Value
	N = 3471	N=3139	N=332	
Age (years), mean (SD)	46.1 (7.8)	46.2 (8.0)	44.6 (5.4)	<0.001
Sleep duration (h), mean (SD)	6.75 (1.21)	6.75 (1.20)	6.75 (1.26)	0.997
DASH score, mean (SD)	21.6 (2.4)	21.5 (2.3)	22.8 (2.4)	<0.001
BMI (kg/m^2), mean (SD)	25.2 (3.3)	25.4 (3.3)	23.8 (3.1)	<0.001
Systolic blood pressure (mmHg), mean (SD)	129.6 (16.6)	130.5 (16.5)	121.8 (14.7)	<0.001
Diastolic blood pressure (mmHg), mean (SD)	82.9 (10.6)	83.4 (10.6)	77.7 (9.8)	<0.001
Fasting blood glucose (mmol/L), mean (SD)	6.13 (1.37)	6.17 (1.39)	5.76 (1.13)	<0.001
Total cholesterol (mmol/L), mean (SD)	5.15 (0.99)	5.16 (0.99)	5.07 (0.97)	0.135
Triglycerides (mmol/L), mean (SD)	1.69 (1.56)	1.75 (1.60)	1.16 (0.91)	<0.001
HDL-C (mmol/L), mean (SD)	1.31 (0.33)	1.29 (0.32)	1.53 (0.36)	<0.001
LDL-C (mmol/L), mean (SD)	3.25 (0.87)	3.26 (0.87)	3.15 (0.88)	0.030
Age (years), n (%)				<0.001
23–29	144 (4.2)	141 (4.5)	3 (0.9)	
30–39	569 (16.4)	508 (16.2)	61 (18.4)	
40–49	1427 (41.1)	1202 (38.3)	225 (67.8)	
50–60	1331 (38.4)	1288 (41.0)	43 (13.0)	
Marital status, n (%)				0.003
Unmarried	89 (2.6)	87 (2.8)	2 (0.6)	
Married	3288 (94.7)	2974 (94.7)	314 (94.6)	
Other	94 (2.7)	78 (2.5)	16 (4.8)	
Education level, n (%)				0.023
Primary or Middle	1027 (29.6)	950 (30.3)	77 (23.2)	
High school or college	1846 (53.2)	1657 (52.8)	189 (56.9)	
University and above	598 (17.2)	532 (17.0)	66 (19.9)	
Smoking status, n (%)				<0.001
Never	1435 (41.3)	1137 (36.2)	298 (89.8)	
Ever	233 (6.7)	227 (7.2)	6 (1.8)	
Current (< 11 pack–years)	657 (18.9)	649 (20.7)	8 (2.4)	
Current (11–20 pack–years)	352 (10.1)	341 (10.9)	11 (3.3)	
Current (> 20 pack–years)	794 (22.9)	785 (25.0)	9 (2.7)	
Drinking status, n (%)				<0.001
Never	2014 (58.0)	1708 (54.4)	306 (92.2)	
Ever	118 (3.4)	112 (3.6)	6 (1.8)	

Current (<140 g/week)	986 (28.4)	969 (30.9)	17 (5.1)	
Current (≥140 g/week)	353 (10.2)	350 (11.1)	3 (0.9)	
Physical activity, n (%)				0.598
Low	37 (1.0)	35 (1.1)	2 (0.6)	
Moderate	246 (7.1)	220 (7.0)	26 (7.8)	
High	3188 (91.9)	2884 (91.9)	304 (91.6)	
Sleep duration (h), n (%)				0.531
<6	433 (12.5)	388 (12.4)	45 (13.6)	
≥6	3038 (87.5)	2751 (87.6)	287 (86.5)	
Insomnia, n (%)				0.435
No	2264 (65.2)	2041 (65.0)	223 (67.2)	
Yes	1207 (34.8)	1098 (35.0)	109 (32.8)	
BMI(kg/m^2), n (%)				<0.001
<25	1732 (49.9)	1501 (47.8)	231 (69.6)	
25–30	1467 (42.3)	1381 (44.0)	86 (25.9)	
≥30	272 (7.8)	257 (8.2)	15 (4.5)	
Hypertension, n (%)				<0.001
No	2344 (67.5)	2075 (66.1)	269 (81.0)	
Yes	1127 (32.5)	1064 (33.9)	63 (19.0)	
Diabetes, n (%)				<0.001
No	3002 (86.5)	2687 (85.6)	315 (94.9)	
Yes	469 (13.5)	452 (14.4)	17 (5.1)	
Dyslipidemia, n (%)				<0.001
No	2072 (59.7)	1089 (57.6)	263 (79.2)	
Yes	1399 (40.3)	1330 (42.4)	69 (20.8)	
Drug use (yes), n (%)	314 (9.1)	301 (9.6)	13 (3.9)	<0.001
Shift work, n(%)				0.001
Never	679 (19.6)	595 (19.0)	84 (25.3)	
Ever	730 (21.0)	649 (20.7)	81 (24.4)	
Current	2062 (59.4)	1895 (60.4)	167 (50.3)	
Cooling protection, n (%)	2600 (74.9)	2353 (75.0)	247 (74.4)	0.822
Wear earplugs, n (%)	1653 (47.6)	1478 (47.1)	175 (52.7)	0.051
Exposure status, n (%)				<0.001
Non-exposure	1036 (29.9)	893 (28.5)	143 (43.1)	
Heat stress only	885 (25.5)	843 (26.9)	42 (12.6)	
Noise only	797 (23.0)	689 (22.0)	108 (32.5)	
Heat stress + Noise	753 (21.7)	714 (22.7)	39 (11.8)	

Carotid plaque, n (%)	1045 (30.1)	1004 (32.0)	41 (12.4)	<0.001

Values are expressed as the mean (SD) or number (%); p values were from Pearson's χ^2 test for categorical variables and t-test for continuous variables. DASH, dietary approaches to stop hypertension; BMI, body mass index; HDL-C, high density lipoprotein cholesterol; LDL-C, low density lipoprotein cholesterol.

Table S2 Basic characteristics of participants according to occupational exposure status

Variables	Total	Non-exposure	Heat only	Noise only	Heat +Noise	*p*-Value
	N = 3471	N=1036	N=885	N=797	N=753	
Sex (male), n (%)	3139 (90.4)	893 (86.2)	843 (95.3)	689 (86.5)	714 (94.8)	<0.001
Age (years), mean (SD)	46.1 (7.8)	47.0 (7.5)	46.2 (8.4)	45.3 (7.9)*	45.4 (7.2)*	<0.001
Sleep duration (h), mean (SD)	6.75 (1.21)	6.72 (1.25)	6.59 (1.24)	6.87 (1.17) #	6.86 (1.12) #	<0.001
DASH score, mean (SD)	21.6 (2.4)	21.6 (2.3)	21.6 (2.3)	21.6 (2.6)	21.6 (2.5)	0.910
BMI (kg/m^2), mean (SD)	25.2 (3.3)	25.1 (3.3)	25.3 (3.4)	25.0 (3.3)	25.5 (3.3)	0.009
Systolic blood pressure (mmHg), mean (SD)	129.6 (16.6)	129.9 (17.4)	129.6 (15.4)	129.2 (17.2)	129.8 (16.1)	0.807
Diastolic blood pressure (mmHg), mean (SD)	82.9 (10.6)	83.3 (10.9)	82.6 (10.4)	82.5 (11.1)	83.0 (9.9)	0.324
Fasting blood glucose (mmol/L), mean (SD)	6.13 (1.37)	6.21 (1.55)	6.14 (1.33)	6.09 (1.41)	6.05 (1.10)	0.108
Total cholesterol (mmol/L), mean (SD)	5.15 (0.99)	5.12 (0.93)	5.15 (0.98)	5.11 (1.00)	5.23 (1.05)	0.048
Triglycerides (mmol/L), mean (SD)	1.69 (1.56)	1.68 (1.52)	1.74 (1.59)	1.63 (1.47)	1.72 (1.66)	0.471
HDL-C (mmol/L), mean (SD)	1.31 (0.33)	1.32 (0.33)	1.31 (0.33)	1.32 (0.33)	1.29 (0.33)	0.325
LDL-C (mmol/L), mean (SD)	3.25 (0.87)	3.21 (0.83)	3.28 (0.88)	3.21 (0.88)	3.32 (0.91)	0.026
Age (years), n (%)						<0.001
23–29	144 (4.2)	37 (3.6)	56 (6.3)	41 (5.1)	10 (1.3)	
30–39	569 (16.4)	128 (12.4)	126 (14.2)	140 (17.6)	175 (23.2)	
40–49	1427 (41.1)	432 (41.7)	334 (37.7)	355 (44.5)	306 (40.6)	
50–60	1331 (38.4)	439 (42.4)	369 (41.7)	261 (32.8)	262 (34.8)	
Marital status, n (%)						0.017
Unmarried	89 (2.6)	25 (2.4)	32 (3.6)	26 (3.3)	6 (0.8)	
Married	3288 (94.7)	983 (94.9)	829 (93.7)	752 (94.4)	724 (96.2)	
Other	94 (2.7)	28 (2.7)	24 (2.7)	19 (2.4)	23 (3.1)	
Education level, n (%)						<0.001
Primary or Middle	1027 (29.6)	353 (34.1)	238 (26.9)	219 (27.5)	217 (28.8)	
High school or college	1846 (53.2)	531 (51.3)	512 (57.9)	402 (50.4)	401 (53.3)	
University and above	598 (17.2)	152 (14.7)	135 (15.3)	176 (22.1)	135 (17.9)	
Smoking status, n (%)						<0.001
Never	1435 (41.3)	447 (43.2)	321 (36.3)	375 (47.1)	292 (38.8)	
Ever	233 (6.7)	73 (7.1)	51 (5.8)	52 (6.5)	57 (7.6)	

Current (< 11 pack–years)	657 (18.9)	177 (17.1)	191 (21.6)	141 (17.7)	148 (19.7)	
Current (11–20 pack–years)	352 (10.1)	89 (8.6)	90 (10.2)	72 (9.0)	101 (13.4)	
Current (> 20 pack–years)	794 (22.9)	250 (24.1)	232 (26.2)	157 (19.7)	155 (20.6)	
Drinking status, n (%)						<0.001
Never	2014 (58.0)	605 (58.4)	463 (52.3)	516 (64.7)	430 (57.1)	
Ever	118 (3.4)	37 (3.6)	26 (2.9)	30 (3.8)	25 (3.3)	
Current (<140 g/week)	986 (28.4)	289 (27.9)	286 (32.3)	192 (24.1)	219 (29.1)	
Current (≥140 g/week)	353 (10.2)	105 (10.1)	110 (12.4)	59 (7.4)	79 (10.5)	
Physical activity, n (%)						0.139
Low	37 (1.0)	12 (1.2)	8 (0.9)	5 (0.6)	12 (1.6)	
Moderate	246 (7.1)	63 (6.1)	64 (7.2)	52 (6.5)	67 (8.9)	
High	3188 (91.9)	961 (92.8)	813 (91.9)	740 (92.9)	674 (89.5)	
Sleep duration (h), n (%)						0.005
<6	433 (12.5)	141 (13.6)	132 (14.9)	84 (10.5)	76 (10.1)	
≥6	3038 (87.5)	895 (86.4)	753 (85.1)	713 (89.5)	677 (89.9)	
Insomnia, n (%)						0.010
No	2264 (65.2)	676 (65.3)	607 (68.6)	485 (60.9)	496 (65.9)	
Yes	1207 (34.8)	360 (34.8)	278 (31.4)	312 (39.2)	257 (34.1)	
BMI(kg/m^2), n (%)						0.040
<25	1732 (49.9)	542 (52.3)	426 (48.1)	422 (53.0)	342 (45.4)	
25–30	1467 (42.3)	419 (40.4)	390 (44.1)	316 (39.6)	342 (45.4)	
≥30	272 (7.8)	75 (7.2)	69 (7.8)	59 (7.4)	69 (9.2)	
Hypertension, n (%)						0.098
No	2344 (67.5)	671 (64.8)	613 (69.3)	536 (67.3)	524 (69.6)	
Yes	1127 (32.5)	365 (35.2)	272 (30.7)	261 (32.8)	229 (30.4)	
Diabetes, n (%)						0.010
No	3002 (86.5)	871 (84.1)	760 (85.9)	669 (87.7)	672 (89.2)	
Yes	469 (13.5)	165 (15.9)	125 (14.1)	98 (12.3)	81 (10.8)	
Dyslipidemia, n (%)						0.027
No	2072 (59.7)	627 (60.5)	534 (60.3)	496 (62.2)	415 (55.1)	
Yes	1399 (40.3)	409 (39.5)	351 (40.0)	301 (37.8)	338 (44.9)	
Drug use (yes), n (%)	314 (9.1)	119 (11.5)	70 (7.9)	75 (9.4)	50 (6.6)	0.003
Shift work, n(%)						<0.001
Never	679 (19.6)	256 (24.7)	199 (22.5)	179 (22.5)	45 (6.0)	
Ever	730 (21.0)	282 (27.2)	220 (24.9)	148 (18.6)	80 (10.6)	

Current	2062 (59.4)	498 (48.1)	466 (52.7)	470 (59.0)	628 (83.4)	
Cooling protection, n (%)	2600 (74.9)	757 (73.1)	662 (74.8)	576 (72.3)	605 (80.4)	<0.001
Wear earplugs, n (%)	1653 (47.6)	388 (29.9)	305 (25.5)	510 (64.0)	450 (59.8)	<0.001
Carotid plaque, n (%)	1045 (30.1)	268 (25.9)	263 (29.7)	233 (29.2)	281 (37.2)	<0.001

* Compared with non-exposure group;

# Compared with heat only group;

Values are expressed as the mean (SD) or number (%); p values were from Pearson's χ^2 test for categorical variables and analysis of variance (ANOVA) for continuous variables. Bonferroni method was used to compare the mean of multiple groups, and the adjusted test level was 0.0083.

DASH, dietary approaches to stop hypertension; BMI, body mass index; HDL-C, high density lipoprotein cholesterol; LDL-C, low density lipoprotein cholesterol.

Table S3 Interactions between occupational heat stress and noise on odds of carotid plaque

Interaction terms	Total
Multiplicative interaction, OR (95% CI)	1.014 (0.863 to 1.192)
Additive interaction [a]	
Relative excess risk due to interaction, RERI (95% CI)	0.211 (-0.267 to 0.689)
Attributable proportion due to interaction, AP (95% CI)	0.106 (-0.127 to 0.339)
Synergy index, S (95% CI)	1.272 (0.709 to 2.282)

OR, odds ratio; CI, confidence intervals; RERI, relative excess risk due to interaction; AP, attributable proportion due to interaction;

[a] If there is no biological interaction, RERI and AP are equal to 0 and S is equal to 1.

Adjusted for age (continuous variable), sex (male, female), marital status (unmarried, married, other), educational level (primary or middle, high school or college, university and above), BMI (<25 kg/m^2, 25–30 kg/m^2, ≥30 kg/m^2), smoking status (never, ever, <11 pack–years, 11–20 pack–years, >20 pack–years), drinking status (never, ever, <140 g/week, ≥140 g/week), DASH score (continuous variable), physical activity (low, moderate, high), sleep duration (<6 hours, ≥6 hours), insomnia (no/yes), hypertension (no/yes), diabetes (no/yes), dyslipidemia (no/yes), medication use (no/yes), shift work (never, ever, current), cooling protection (no/yes), wear earplugs (no/yes).

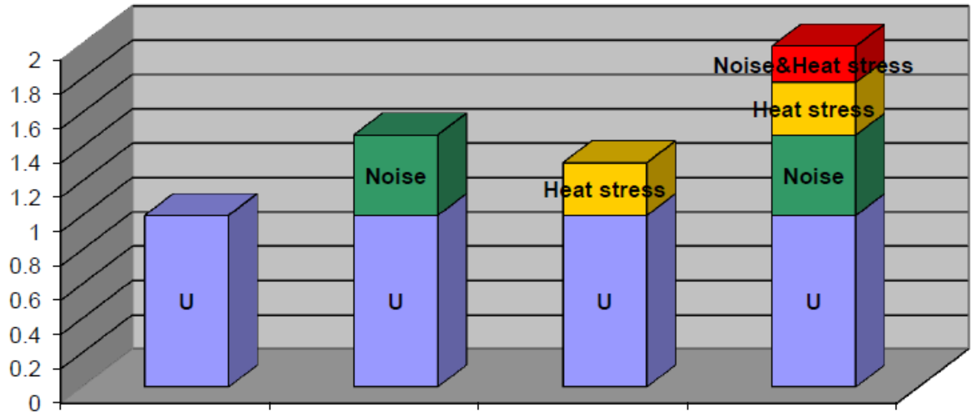

Figure S1 Relative risk with contributions from different exposure categories marked in additive interaction. U is the common reference category.

Table S4 Multivariate adjusted ORs between carotid plaque and different occupational exposure status.

Exposure status	Model 1	Model 2	Model 3
Non exposure	1.00	1.00	1.00
Heat stress only	1.26 (1.02 to 1.56)	1.33 (1.07 to 1.66)	1.32 (1.06 to 1.65)
Noise only	1.43 (1.15 to 1.78)	1.49 (1.19 to 1.87)	1.45 (1.15 to 1.83)
Heat stress + Noise	1.96 (1.58 to 2.44)	2.12 (1.69 to 2.65)	1.91 (1.52 to 2.41)

Model 1: Adjusted for sex and age group (23–29 years, 30–39 years, 40–49 years, 50–60 years) based on Table 2. Model 2: Adjusted for Model 1 + marital status (unmarried, married, other), educational level (primary or middle, high school or college, university and above), BMI (<25 kg/m^2, 25–30 kg/m^2, ≥30 kg/m^2), smoking status (never, ever, <11 pack–years, 11–20 pack–years, >20 pack–years), drinking status (never, ever, <140 g/week, ≥140 g/week), DASH score (continuous variable), physical activity (low, moderate, high), sleep duration (<6 hours, ≥6 hours), insomnia (no/yes), hypertension (no/yes), diabetes (no/yes), dyslipidemia (no/yes), medication use (no/yes). Model 3: Adjusted for Moodel 2 + shift work (never, ever, current), cooling protection (no/yes), wear earplugs (no/yes).

The Omnibus Tests of Model Coefficients of the model were generally meaningful with P<0.01, the P values of Hosmer and Lemeshow tests for the model were 0.638, 0.190 and 0.861, respectively, indicating a high goodness of fit. The variance inflation factor (VIF) of the covariates in the model ranged from 1.006 to 1.488, without multicollinearity.

Table S5 Multivariate adjusted ORs between carotid plaque and different occupational exposure status.

**Exposure status**	**Estimate**	**Standard Error**	**Wald Chi-Square**	**OR (95%CI)**	***p*-Value**
Non exposure	-	-	-	1.00	-
Heat stress only	-0.042	0.072	0.345	1.32 (1.06 to 1.65)	0.557
Noise only	0.047	0.075	0.389	1.45 (1.15 to 1.83)	0.533
Heat stress + Noise	0.318	0.074	18.277	1.90 (1.50 to 2.40)	<0.001

Adjusted for age group (23–29 years, 30–39 years, 40–49 years, 50–60 years), sex (male, female), marital status (unmarried, married, other), educational level (primary or middle, high school or college, university and above), BMI (<25 kg/m^2, 25–30 kg/m^2, ≥30 kg/m^2), smoking status (never, ever, <11 pack–years, 11–20 pack–years, >20 pack–years), drinking status (never, ever, <140 g/week, ≥140 g/week), DASH score (continuous variable), physical activity (low, moderate, high), sleep duration (<6 hours, ≥6 hours), insomnia (no/yes), hypertension (no/yes), diabetes (no/yes), dyslipidemia (no/yes), medication use (no/yes), shift work (never, ever, current), cooling protection (no/yes), wear earplugs (no/yes).

The Omnibus Tests of Model Coefficients of the model was generally meaningful with P<0.01, the P value of Hosmer and Lemeshow tests for the model was 0.859, indicating a high goodness of fit. The variance inflation factor (VIF) of the covariates in the model ranged from 1.010 to 1.487, without multicollinearity.

Table S6 Multivariate adjusted ORs between carotid plaque and different occupational exposure status.

**Variables**	**Total**	**Estimate**	**Wald Chi-Square**	**OR (95%CI)**	***p*-Value**
Sex, n (%)					
Male	3139 (90.4)			1.00	
Female	332 (9.6)	-0.555	8.595	0.57 (0.40 to 0.83)	0.003
Age (years), mean (SD)	46.1 (7.8)	0.114	205.461	1.12 (1.10 to 1.14)	<0.001

DASH score, mean (SD)	21.6 (2.4)	0.010	0.324	1.01 (0.98 to 1.05)	0.569
Marital status, n (%)					
Unmarried	89 (2.6)			1.00	
Married	3288 (94.7)	0.123	0.436	1.01 (0.39 to 2.64)	0.509
Other	94 (2.7)	-0.234	0.970	0.71 (0.24 to 2.09)	0.325
Education level, n (%)					
Primary or Middle	1027 (29.6)			1.00	
High school or college	1846 (53.2)	-0.079	1.738	0.86 (0.72 to 1.03)	0.187
University and above	598 (17.2)	0.002	0.001	0.93 (0.69 to 1.25)	0.982
Smoking status, n (%)					
Never	1435 (41.3)			1.00	
Ever	233 (6.7)	-0.287	4.746	0.84 (0.60 to 1.19)	0.029
Current (< 11 pack–years)	657 (18.9)	0.054	0.354	1.18 (0.93 to 1.51)	0.552
Current (11–20 pack–years)	352 (10.1)	0.314	7.569	1.54 (1.14 to 2.07)	0.006
Current (> 20 pack–years)	794 (22.9)	0.035	0.185	1.16 (0.93 to 1.45)	0.667
Drinking status, n (%)					
Never	2014 (58.0)			1.00	
Ever	118 (3.4)	0.229	1.972	1.73 (1.12 to 2.67)	0.160
Current (<140 g/week)	986 (28.4)	-0.082	0.988	1.27 (1.05 to 1.53)	0.320
Current (≥140 g/week)	353 (10.2)	0.170	2.536	1.63 (1.25 to 2.13)	0.111
Physical activity, n (%)					
Low	37 (1.0)			1.00	
Moderate	246 (7.1)	0.073	0.175	1.40 (0.59 to 3.36)	0.676
High	3188 (91.9)	0.192	1.601	1.58 (0.69 to 3.61)	0.206
Sleep duration (h), n (%)					
<6	433 (12.5)			1.00	
≥6	3038 (87.5)	0.200	2.641	1.22 (0.96 to 1.55)	0.104
Insomnia, n (%)					
No	2264 (65.2)			1.00	
Yes	1207 (34.8)	0.326	14.358	1.39 (1.17 to 1.64)	<0.001
BMI(kg/m^2), n (%)					
<25	1732 (49.9)			1.00	
25–30	1467 (42.3)	-0.093	1.932	0.94 (0.79 to 1.11)	0.165
≥30	272 (7.8)	0.119	1.366	1.16 (0.85 to 1.59)	0.243
Hypertension, n (%)					

No	2344 (67.5)			1.00	
Yes	1127 (32.5)	0.258	7.722	1.29 (1.08 to 1.55)	0.006
Diabetes, n (%)					
No	3002 (86.5)			1.00	
Yes	469 (13.5)	0.365	10.209	1.44 (1.15 to 1.80)	0.001
Dyslipidemia, n (%)					
No	2072 (59.7)			1.00	
Yes	1399 (40.3)	0.368	18.468	1.45 (1.22 to 1.71)	<0.001
Drug use, n (%)					
No	3157 (90.9)			1.00	
Yes	314 (9.1)	0.372	6.813	1.45 (1.10 to 1.92)	0.009
Shift work, n (%)					
Never	679 (19.6)			1.00	
Ever	730 (21.0)	0.142	3.986	1.66 (1.27 to 2.17)	0.046
Current	2062 (59.4)	0.224	14.037	1.81 (1.43 to 2.28)	<0.001
Cooling protection, n (%)					
No	871 (25.1)			1.00	
Yes	2600 (74.9)	-0.033	0.113	0.97 (0.80 to 1.17)	0.737
Wear earplugs, n (%)					
No	1818 (52.4)			1.00	
Yes	1653 (47.6)	0.134	2.376	1.14 (0.96 to 1.36)	0.123
Exposure status, n (%)					
Non-exposure	1036 (29.9)			1.00	
Heat stress only	885 (25.5)	-0.062	0.729	1.32 (1.06 to 1.65)	0.393
Noise only	797 (23.0)	0.052	0.467	1.48 (1.17 to 1.87)	0.494
Heat stress + Noise	753 (21.7)	0.352	21.947	2.00 (1.58 to 2.53)	<0.001

Adjusted for age (continuous variable), sex (male, female), marital status (unmarried, married, other), educational level (primary or middle, high school or college, university and above), BMI (<25 kg/m^2, 25–30 kg/m^2, ≥30 kg/m^2), smoking status (never, ever, <11 pack–years, 11–20 pack–years, >20 pack–years), drinking status (never, ever, <140 g/week, ≥140 g/week), DASH score (continuous variable), physical activity (low, moderate, high), sleep duration (<6 hours, ≥6 hours), insomnia (no/yes), hypertension (no/yes), diabetes (no/yes), dyslipidemia (no/yes), medication use (no/yes), shift work (never, ever, current), cooling protection (no/yes), wear earplugs (no/yes).

The Omnibus Tests of Model Coefficients of the model was generally meaningful with P<0.01, the P value of Hosmer and Lemeshow tests for the model was 0.857, indicating a high goodness of fit. The variance inflation factor (VIF) of the covariates in the model ranged from 1.010 to 1.489, without multicollinearity.

References

1. Li X, Cui S, Wu J, Wang L, Yuan J: **Job category differences in the prevalence and associated factors of insomnia in steel workers in China**. *International journal of occupational medicine and environmental health* 2020, **33**(2):215-233.
2. Kraïm-Leleu M, Lesage F-X, Drame M, Lebargy F, Deschamps F: **Occupational Risk Factors for COPD: A Case-Control Study**. *PloS one* 2016, **11**(8):e0158719.
3. Millwood IY, Walters RG, Mei XW, Guo Y, Yang L, Bian Z, Bennett DA, Chen Y, Dong C, Hu R *et al*: **Conventional and genetic evidence on alcohol and vascular disease aetiology: a prospective study of 500 000 men and women in China**. *Lancet (London, England)* 2019, **393**(10183):1831-1842.
4. Maskarinec G, Lim U, Jacobs S, Monroe KR, Ernst T, Buchthal SD, Shepherd JA, Wilkens LR, Marchand LL, Boushey CJ: **Diet Quality in Midadulthood Predicts Visceral Adiposity and Liver Fatness in Older Ages: The Multiethnic Cohort Study**. *Obesity (Silver Spring, Md)* 2017, **25**(8):1442-1450.
5. Fung TT, Chiuve SE, McCullough ML, Rexrode KM, Logroscino G, Hu FB: **Adherence to a DASH-style diet and risk of coronary heart disease and stroke in women**. *Archives of internal medicine* 2008, **168**(7):713-720.
6. Celis-Morales CA, Perez-Bravo F, Ibañez L, Salas C, Bailey MES, Gill JMR: **Objective vs. self-reported physical activity and sedentary time: effects of measurement method on relationships with risk biomarkers**. *PloS one* 2012, **7**(5):e36345.
7. Soldatos CR, Dikeos DG, Paparrigopoulos TJ: **Athens Insomnia Scale: validation of an instrument based on ICD-10 criteria**. *Journal of psychosomatic research* 2000, **48**(6):555-560.
